# Pigs fed camelina meal increase hepatic gene expression of cytochrome 8b1, aldehyde dehydrogenase, and thiosulfate transferase

**DOI:** 10.1186/2049-1891-5-1

**Published:** 2014-01-03

**Authors:** William Jon Meadus, Pascale Duff, Tanya McDonald, William R Caine

**Affiliations:** 1 AAFC-Lacombe, 6000 C&E Trail, Lacombe, AB, Canada T4L 1 W1; 2 School of Innovation, Olds College, 4500-50th St., Olds, AB, Canada T4H 1R6; 3 Caine Research Consulting, Box #1124, Nisku, AB, Canada T9E 8A8

**Keywords:** Camelina meal, Gene expression, Glucosinolates, Pig liver

## Abstract

*Camelina sativa* is an oil seed crop which can be grown on marginal lands. Camelina seed oil is rich in omega-3 fatty acids (>35%) and γ-tocopherol but is also high in erucic acid and glucosinolates. Camelina meal, is the by-product after the oil has been extracted. Camelina meal was fed to 28 d old weaned pigs at 3.7% and 7.4% until age 56 d. The camelina meal supplements in the soy based diets, improved feed efficiency but also significantly increased the liver weights. Gene expression analyses of the livers, using intra-species microarrays, identified increased expression of phase 1 and phase 2 drug metabolism enzymes. The porcine versions of the enzymes were confirmed by real time PCR. Cytochrome 8b1 (CYP8B1), aldehyde dehydrogenase 2 (Aldh2), and thiosulfate transferase (TST) were all significantly stimulated. Collectively, these genes implicate the camelina glucosinolate metabolite, methyl-sulfinyldecyl isothiocyanate, as the main xeniobiotic, causing increased hepatic metabolism and increased liver weight.

## Background

*Camelina sativa*, a member of the family *Brassicaceae* is related to rapeseed [1]. It has commercial value as an oil seed crop for biofuels and biolubricants and can be grown on marginal lands [2]. Camelina seed has an oil content of over 40% (dry weight) and this oil is high in omega-3 fatty acids, gamma tocopherol [3] but also in the monounsaturated omega-9 fatty acid, erucic acid (C22:1 ω-9) [4]. Typical seed crushers will extract the oil content from the seed down to 4% (dry weight) leaving the meal. The Camelina meal still has a problem because, after the oil has been extracted, it can have a total glucosinolate content of ~ 24 μmol/g [5]. In canola meal, when total glucosinolate content is higher than 15 μmol/g of feed, it will reduce feed intake and growth in finishing pigs [6,7].

Glucosinolates are considered bitter to humans [8]. Chickens [9], fish (trout) [10] and pigs [4] will initially show reduced feed intakes depending on the dosage. The maximum recommended dose of glucosinolates for monogastric animals, such as swine, is approximately 2 umoles/g of feed. The glucosinolates are metabolized by endogenous plant enzymes called myrosinase or β-thioglucosidases from gut bacteria, into biologically active compounds, isothiocyanates, indoles and nitriles [8]. Higher doses of the glucosinolate metabolite, thiocyanate, can affect the transport of iodine to thyroid [11]. Glucosinolate metabolic products are mainly associated with the induction of Phase I and Phase 2 biotransformation enzymes [12]. Phase 1 enzymes catalyse a variety of hydrolytic, oxidative and reductive reactions, including the cytochrome P450s involved in metabolizing xenobiotics and toxins [13]. Phase 2 enzymes, such as glutathione S-transferase and UDP-glucuronyl transferase, form conjugation products with xenobiotics and are readily excreted [14]. When feeding xenobiotics, liver is typically the most responsive tissue for phase 1 and 2 expression. However, the phase 1 and 2 enzymes are expressed almost ubiquitously throughout the body, in multiple species, including humans.

Glucosinoates metabolites from camelina, rapeseed and canola differ slightly in the type and quantity. Glucosinolates metabolites from rapeseed and Canadian canola (Brassica napsis) are predominately progoitrin (2-hydroxy-3-butenyl) and gluconapin (3-butenyl) structure [15]. Glucosinolates from camelina are predominantly glucocamelina, which is metabolized into 10-methylsulphinyldecyl isothiocyanate [16]. The structure of 10-methylsulfinyldecyl is closely analogous to sulforaphane which is common to cruciferous vegetables such as broccoli, mustard and cabbage and may be protective against cancer and cardiovascular disease [17]. The present study was undertaken as a preliminary investigation of pigs receiving camelina meal as alternative feed source and as an animal model to assess the potential health benefits.

## Methods

### Animals and feed

The feeding trial was performed in Lacombe Research center piggery, under the supervision of trained Agriculture and Agri-Food staff that monitored the animals in accordance with guidelines of the Canadian Council on Animal Care. The pig breed in the study was a Large White X Duroc (Hypor Inc, Regina, SK). The camelina meal was provided by Canpresso (Midland, SK, Canada). The HPLC analysis of camelina for glucocamelinin, 10-methyl-sulfinyldecyl, and 11-methyl-sulfinyldecyl content was made using method AK 1-92 of the American Oil Chemist Society (AOCS) by Bioprofile Testing Labs (St. Paul, MN, USA) (Table 1). The molecular weight of glucocamelinin C_18_H_35_O_10_S_3_N is estimated to be 521.65 g/mol; therefore the glucocamelinin content of 22.84 µmol/kg meal, is equivalent to 12.36 g/kg of camelina meal. The feeding trial was composed of three groups fed either, the control (CON) diet, the 3.7% camelina meal supplemented LOW diet, or the 7.4% camelina meal supplemented HIGH diet, for 28 d (Table 2). The soy concentrate (Cargill, Elk River, MN) and corn starch based grower diets were balanced with canola oil to be iso-caloric but not iso-nitrogenous. Compositions of the diets were calculated to meet the Nutrient requirements of swine [18]. The HIGH diet had slightly more estimated crude protein 144.0 g/kg than the control diet at 141.3 g/kg (Table 2). The piglets were started on standard weaner diet [18] prepared by Wetaskiwin Co-op Feeds (Wetaskiwin, Alberta) until the feeding trial test diets, which were started, 2-wk post weaning, at age of 28 d, on 27 barrows, weighing 12.7 ±1.73 kg. The piglets were held individually in metabolic crates penned on concrete floor with slatted sections. The feed was provided *ad libitum* and they had full access to drinking water for the 28 d trial. Their weights and feed were monitored daily; until they reached 56 d. Pigs were euthanized in accordance to CCAC guidelines [19]. Organ tissues were removed *post mortem*, weighed (Table 3) and stored at -20°C.

**Table 1:** Chemical analysis of camelina meal’s crude fat, crude protein and glucosinolate content

**Item**^**1**^**, g/kg**	
Dry matter	928
Crude protein	363
Crude fat	143
Calcium	2.1
Phosphorus	9.6
Glucosinolates, μmol/g	23.79
Glucocamelinin	22.84
10-methyl-sulfinyldecyl	0.95

^1^Dry matter basis.

**Table 2:** Composition of the experimental cornstarch soy-concentrate-based diets without (Control) or with Low (37 g/kg) or High (74 g/kg) levels of added camelina meal

**Diet**	**Control**	**Low camelina**	**High camelina**
Ingredients^1^, g/kg			
Corn starch	639.1	634.1	629.2
Soy concentrate	200.0	183.0	166.0
Camelina meal	-	37.0	74.0
Canola oil	50.0	44.7	39.4
Solka-floc (cellulose)	60.0	50.3	40.6
Dicalcium phosphate (21%)	14.0	14.0	14.0
Sodium chloride	2.7	2.7	2.7
Magnesium oxide (56%)	2.3	2.3	2.3
Sodium bicarbonate	2.0	2.0	2.0
Calcium carbonate	12.0	12.0	12.0
Trace mineral mix	1.0	1.0	1.0
Choline chloride (60%)	1.0	1.0	1.0
Selenium (1,000 mg/kg)	0.5	0.5	0.5
ADE vitamin mix^2^	0.7	0.7	0.7
Vitamin E (1,000 IG/g)	0.2	0.2	0.2
Lysine hydrochloride	5.5	5.5	5.5
DL-Methionine	3.0	3.0	3.0
L-Threonine	3.0	3.0	3.0
Chromic oxide	3.0	3.0	3.0
Calculated contents, g/kg^1^			
ME, kcal/kg^3^	3,754	3,764	3,775
Protein^4^	141.5	144.9	146.3
Dry matter (DM)	932	926	926
Total glucosinolates, μmol/kg	-	0. 88	1.76

^1^As-fed basis.

^2^ADE vitamin mix: vitamin A (50,000 IU), D3 (5,000 IU), E(50 IU) /g.

^3^Metabolizable Energy; (ME; kcal kg^-1^) calculated based on values, as follows: 3985, cornstarch; 8410, canola and camelina oil; 3250, soy concentrate; 2717, camelina meal.

^4^Protein calculated based on values, as follows: 65% soy concentrate + 36.3% camelina meal + lysine + methionine + threonine.

**Table 3:** Daily feed intake, average daily gain, feed conversion efficiency and organ tissue weights of pigs after a 28 d trial of cornstarch soy-concentrate-based diets without (Control) or with Low (3.7 g/kg) or High (7.4 g/kg) levels of added camelina meal

**Diet**	**Control**	**Low camelina**	**High camelina**	**SEM**^**1**^	***P***
*Item*					
Start weight (kg)	13.3	12.5	12.3	0.7	0.39
Final weight (kg)	17.13	17.05	17.05	0.8	0.99
Average daily gain (g/d)	185.1	236.8	233.8	17.7	0.10
Average feed intake (g/d)	605.9	559.2	518.1	18.1	0.06
Feed conversion (feed/gain)	3.72^a^	2.37^b^	2.32^b^	0.3	0.04
*Organ tissue, g*					
Liver	346.4^b^	418.4^a^	421.7^a^	19.0	0.02
Heart	82.8	90.2	89.0	3.0	0.21
Spleen	35.4	40.1	43.3	2.7	0.14
Thyroid	19.4	17.7	15.9	2.8	0.48

^1^Standard error of the mean (*n* = 27).

^a,b^Values in the same row with different superscripts differ (*P* < 0.05).

### RNA microarray analysis and quantitative PCR

Total pig liver RNA was examined for gene expression changes by microarray analysis using, the Rat Drug metabolism: phase 1 array (PARN-068) and the Human Drug Metabolism: phase 2 array (PAHS-069) (SABioscience /Qiagen, Mississauga, ON). Briefly, Liver samples were collected from the pigs within 10 min post slaughter and stored at -80°C in RNAlater (Qiagen). Total RNA was extracted from the livers (100 mg) by homogenizing in 5 moles/L guanidium isothiocyanate and then binding on silica columns for DNase treatment and washing before collecting the RNA in water according to the manufacturer’s methods of the Aurum Total RNA fatty and fibrous tissue kit (BioRad, Mississauga, Ontario). The quality and quantity of the RNA was assessed spectrophotometrically at 260 nm, 280 nm and 320 nm. The RNA quality was also checked for good, intact, 28S and 18S rRNA bands by visualization on a 1.0% agarose gel electrophoresis stained with 1 ug/mL ethidium bromide

For microarray analysis, total RNA was collected from the control fed pigs and the 7.4% camelina fed pigs and pooled within the three treatments and was converted to cDNA with a RT^2^ First Strand Kit (Qiagen Canada, Mississauga, ON). The pooled cDNA was tested by microarray analysis, on the Rat drug metabolism: phase 1 array (PARN-068) and the Human Drug Metabolism: phase 2 array (PAHS-069) (SABioscience /Qiagen, Mississauga, ON) using the RT^2^ SYBR Green polymerase chain reaction (PCR) Master mix according to manufacturer’s recommendations on a Mx3000P QPCR real time PCR machine with SYBR gene detection (Agilent Technologies Canada Inc., Mississauga, ON). PCR conditions were hot start at 95°C for 10 min followed by 95°C for 15 sec, 55°C for 15 s, and 60°C for 60 s, for 40 cycles. Quantitative gene analysis was based on comparative 2^-ΔΔ^Ct method using the RT^2^ profiler PCR data analysis program 3.5. (SABiosciences/ Qiagen, Mississauga, ON).

The genes identified by the arrays were used in the search for their closest porcine equivalent in the National Center for Biotechnology Information (NCBI) U.S. National Library of Medicine, GenBank [20] using the Basic Local Alignment Search Tool (Blast) version 2.2.27 program. Primers sequences were selected, using the Primer 3 program v.0.4.0, to amplify the porcine version of the gene transcript [21]. Porcine transcripts were confirmed by sequencing the PCR products on a CEQ8000 machine using a GenomeLab™ DTCS Quick Start dideoxy sequencing kit as per manufacturer’s instructions (Beckman Coulter, Mississauga, ON, Canada).

Total RNA was collected from the animals (n = 27) in the three diets and was used to prepare cDNA. RNA (2ug) was combined with, 0.5 ug oligo-dT, 200 mmoles/L dNTPs and preheated at 65°C for 2 min to denature secondary structures. The mixture was then cooled rapidly to +20°C and then 10 uL 5X RT Buffer, 10 mmoles/L DTT and 200 U MMLV Reverse Transcriptase (Sigma-Aldrich, Oakville, ON, Canada) was added for a total volume of 50 uL. The RT mix was incubated at 37°C for 90 min. then stopped by heating at 95°C for 5 min. The cDNA stock was stored at –20°C. The yield of cDNA was measured according to the PCR signal generated from the internal standard house-keeping gene β-actin amplified from 0.1 μL of the cDNA solution. The volume of each cDNA sample was adjusted to give approximately the same exponential phase PCR signal strength for β-actin after 20 cycles [22]. The primers for the porcine version of the microarray selected genes are shown in (Table 4). The cDNA [100 ng] was used in a RT^2^ SYBR Green master mix with 10 umoles/L of each primer run on Mx300P QPCR machine at 95°C/10 min for hot start followed by 40 cycles of 95°C/30 s, 56°C/30 s and 72^o^C/60 s. Relative gene analysis was based on comparative 2^-ΔΔCt^ method [23]. The reference genes were averaged between internal housekeeping genes, GADPH and β–actin.

**Table 4:** **Forward and reverse *porcine *** **primer sequences and accession numbers of genes used in quantitative RT-PCR analysis**

**Gene**	**Forward primer**	**Reverse primer**	**GenBank accession #**	**cDNA size, bp**
CYP8b1	5′-aagtgggccggctccagtgt-3′	5′-gcccgagccccatggcatag-3′	NM_214426.1	625
Aldh2	5′-gcatcggcatgttgcgccct-3′	5′-ggtaggtccggtcccgctca-3′	NM_001044611.2	374
TST	5′-cgggctcaagggcggtacct-3′	5′-tttgcccacggggcatggac-3′	XM_001926303	435
Gst*mu*5	5′-tcgcccgcaagcacaacatgt-3′	5′-acaagcagtgcaagtccgcct-3′	AK233626.1	453
β-actin	5′-acatcaaggagaagctgtgc-3′	5′-ttggcgtagaggtccttgc-3′	AY550069	256

### Statistical analysis

Animals’ responses to the diets were analyzed using ANOVA followed by Duncan’s test for differences in the group means. The gene analysis data was calculated using the comparative 2^-ΔΔCt^ method and significance between treatment groups was determined using the Student’s t-test. Statistical significance was accepted at *P* < 0.05 and trends were indicated at *P* < 0.1. All data were run on SAS version 9 [24].

## Results and discussion

### Diet

The estimated chemical composition of the camelina meal supplemented diets for crude protein and crude fat are outlined (Tables 1 and 2). The amount of crude protein in the meal was estimated to be ~363 g/kg and the amount of crude fat was 143 g/kg, which is high at ~14%, considering the oil was extracted by cold pressure [6]. The concentration of glucosinolates was measured to be ~23.70 μmol/g in the camelina meal using a chromatography method [25]. This content is significantly higher than the average value of 7.8 μmol glucosinolate /g of meal from modern conventional varieties of canola [15]. However, the estimated final concentration of glucocamelinin in the LOW and HIGH supplemented diets was well within the safe region at 0.82 µmol/kg and 1.63 μmol/kg of feed (Table 2).

Camelina contains a high concentration of the antioxidant, gamma tocopherol 1,000 μg/g, which is substantially higher, relative to other oils seed crops such as, canola and flax [3]. Gamma tocopherol is the most commonly found form of vitamin E in plants [26]. It provides antioxidant protection to the oils and gives a nutty aroma to camelina oil. For humans however, alpha-tocopherol is the preferred form and the only isomer selected by the tocopherol transfer protein (α-TTP) and tocopherol associated protein (α-TAP) in the liver [27]. Gamma tocopherol is absorbed but quickly excreted in the urine.

In addition to glucosinolates, Camelina also contains the monounsaturated omega-9 fatty acid, erucic acid. Erucic acid (C20:1 w-9) is a large component of mustard oil and can account to approximately 5% of camelina seed and is suspected to reduce the palatability of feed for monogastric swine [28]. The allowable amount of erucic acid in canola seed in Canada is set at 2% [29]. Erucic acid content is a large component of mustard oil and its consumption by human is still controversial. It implicated in causing thrombocytopenia but it is also part of Lorenzo’s oil [5] used to the treat adrenoleukodystrophy [6]. The crude fat in meal of camelina after crushing and processing was 14.3% and this fat is expected to contain less than 0.715 g erucic acid/kg of meal. The level of erucic acid in the LOW diet was expected to be 0.026 g/kg feed. In the HIGH diet, the erucic acid content was estimated to be 0.053 g/kg of feed (~ 0.005%), which is below the level expected for any detectable physiological effect [30].

### Animals and weights

Mean initial weights of the pigs were 12.7 ±1.73 kg for the start of trial and at the end of the 28 day trial; the pigs weighed an average of 17.1 ±2.12 kg at 56 d (Table 3). The animals on the camelina meal supplemented diets had a significant improvement in their feed efficiency. This may have been due to an increase in the available lysine protein and metabolizable energy available from the camelina meal supplement; although, the soy concentrate, corn starch based control diet, met NRC [18] recommended lysine levels estimated at ~ 14.6 g/kg feed for 20 kg pigs (Table 2). It may have been also due to a reduced feed intake in the LOW and HIGH diets (Table 3). The pigs did have some problems adjusting to the camelina meal diets. The pigs on HIGH diet did have an initial aversion to the meal, with reduced average daily feed intakes of 518.1 ±70.4 g/d, as compared to the CON diet intake at 605.9 ±111.3 g/d but this was only shown as a trend *P* < 0.1. There was a significant increase in the liver weights from the pigs fed the HIGH diet. The liver weights at the end of the trial was 346.4 g/pig for the CON group, 418.4 g/pig for MED group and 427.7 g/pig for HIGH fed group (Table 3). This indicates extra hepatic activity caused by increasing the camelina meal. The pigs fed the LOW and HIGH camelina meal diet also indicated a reduction in their thyroid weights (Table 3). This may be due to a disruption of iodine absorption and thyroid activity caused by glucoinosinolates in the camelina meal and will have to investigated further; possibly in a longer (>28 d) trial in which the iodine level in the pigs are measured.

### Hepatic gene expression

**Figure 1:**
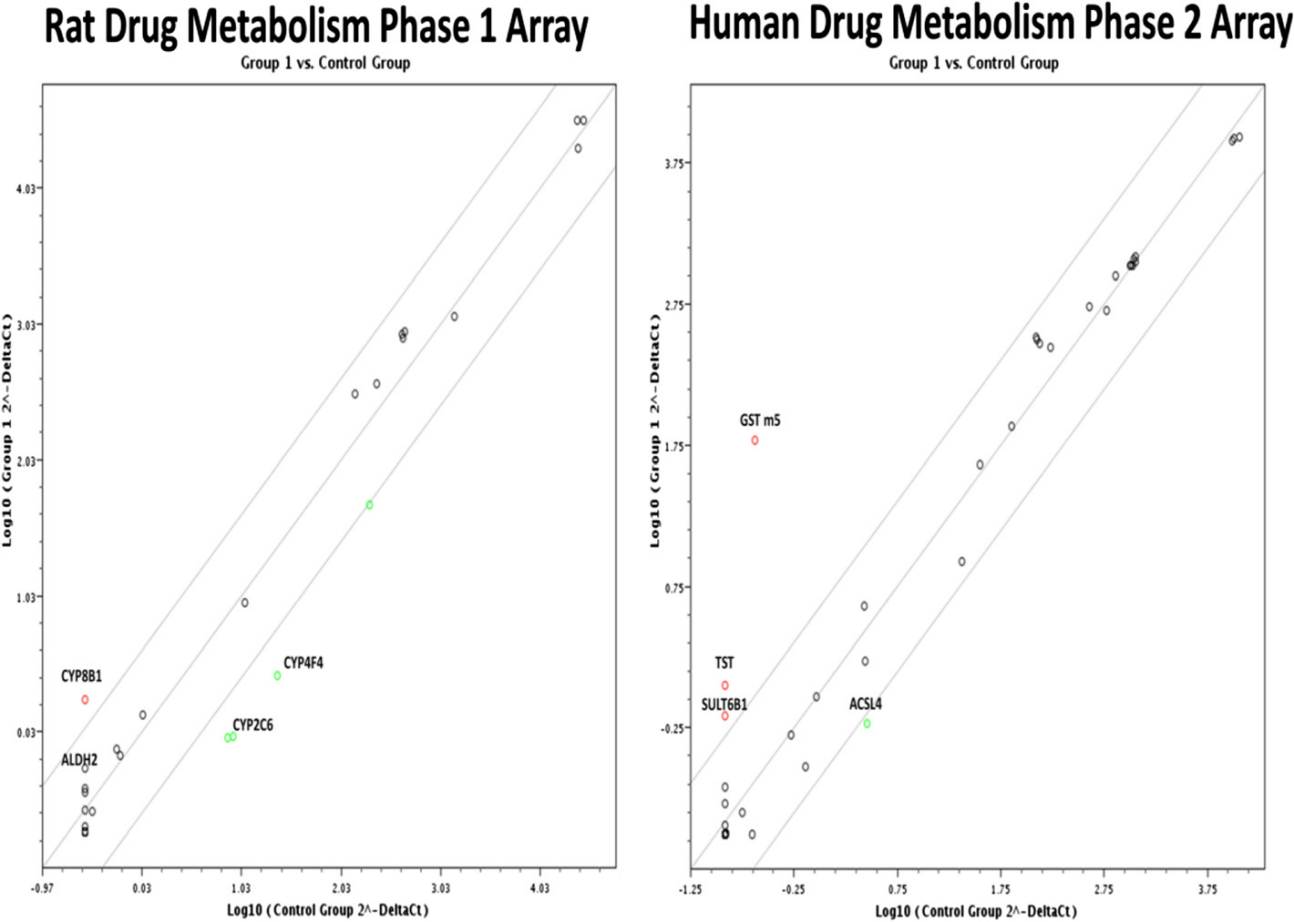
**Total liver RNA examined for gene expression changes by microarray analysis using, the *Rat *drug metabolism: phase 1 array (PARN-068) and the *Human *Drug Metabolism: phase 2 array (PAHS-069).** The genes that were significantly up or down regulated in the microarrays are labelled. The RNA was from a pooled sample of pigs fed the control verses the High 7.4% camelina diet as outlined in the materials and methods.

The total RNA from the pigs livers fed the High level of camelina meal was compared with animal fed the CON diets and was tested on two available microarrays representing 168 genes involved in drug metabolism (Figure 1). The Rat Drug Metabolism Phase I Enzymes RT^2^ Profiler PCR Array PARN – 068A contains 84 genes involved in phase I drug metabolism. Phase I drug metabolism enzymes make compounds more hydrophilic. This array represents genes involved in Phase I drug metabolism reactions including oxidation, reduction, hydrolysis, cyclization, decyclization and members of the Cytochrome P450 enzyme family. The Human Drug Metabolism Phase II Enzymes RT^2^ Profiler™ PCR Array PAHS-069Z, contains 84 genes involved in the enzymatic processes of drug biotransformation. Phase II drug metabolism enzymes catalyze the conjugation of lipophilic compounds with hydrophilic functional groups or moieties to form water-soluble conjugates that can be cleared from cells and from the body. This array represents genes encoding the phase II drug metabolism enzymes catalyzing such reactions as glutathione conjugation, glucuronidation, sulfation, methylation, amino acid conjugation, epoxidation, and esterification. The pig RNA transcripts were expected to be only moderately homologous with humans and rats; therefore, an annealing temperature reduction of 55°C for 15 s was made in the PCR program to overcome some differences between primer sequences.

The rat Drug Metabolism Phase 1 microarray identified the cytochrome P450 -8b1 (Cyp8b1) and the aldehyde dehydrogenase 2 (Aldh2) gene transcription as being stimulated in the pig livers by camelina meal feeding. The human drug metabolism phase 2 array identified glutathione S-transferase mu 5 (GSTM5) and thiosulfate transferase (TST), as being significantly up-regulated > 4-fold (Figure 1) relative to the control livers, as determined by the RT^2^ profiler PCR data analysis program 3.5.

A comparison of the gene sequences identified in the rat array and porcine (Sus scrofa) version of the genes was 87% between the rat Aldh2 GenBank# NM_032416 and the porcine GenBank# NM_001044611.2 and 80% between the rat Cyp8b1 GenBank# NM_031241 and the porcine GenBank# NM_214426.1. Comparison of the genes identified in the human array with porcine (Sus scrofa) mRNA was 86% between the human GSTm5 and the porcine GSTm2 GenBank# AK233626.1 and 89% between the human TST GenBank# NM_003312 and the porcine TST GenBank# XM001926303.2. New porcine specific primers were created based on the aforementioned porcine sequences (Table 4).

**Figure 2:**
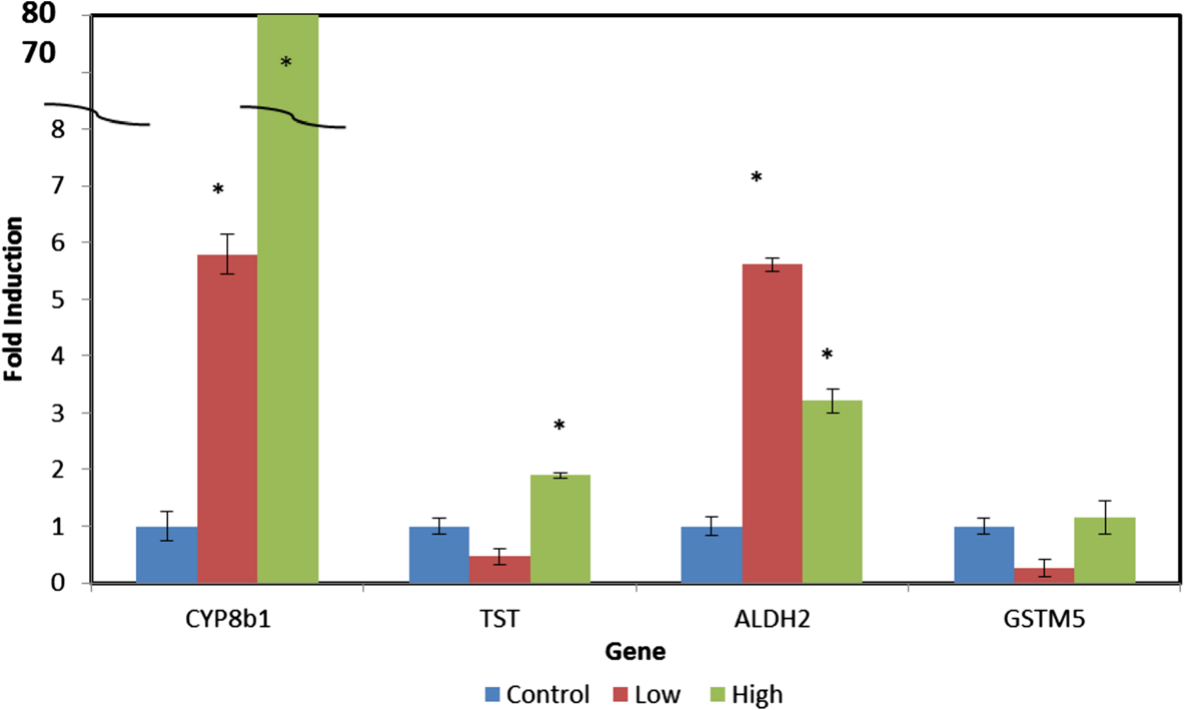
**The effect of camelina meal on the pig liver gene expression level of Cyp8B1, TST, Aldh2, and Gstm5.** Transcripts were determined by real-time PCR with *porcine* specific primers. Pig diets were supplemented with either the Control (0%), Low (3.7%) or High (7.4%) levels of camelina meal for 24d. Error bars represent the standard error of the mean. * indicates *P* > 0.05 relative to the Control using Student’s *t-test*.

The genes investigated directly for mRNA expression by quantitative real-time PCR using porcine specific primers on the livers of the fed pigs LOW and HIGH amounts of camelina meal are shown in Figure 2. Cyp8b1 mRNA was up-regulated approximately 80-fold in the liver tissue of pigs supplemented with High camelina meal. The transcripts for the TST and Aldh2 were increased approximately 1.8 and 3.2 fold but these were only weakly significant (*P* < 0.1). The Gstm5 transcript was not stimulated by adding camelina to their diet.

Cytochrome P450 8B1 (Cyp8b1) is primary a microsomal sterol hydroxylase involved in bile acid formation [31]. Porcine Cyp8b1 will catalyse the hydroxylation of cholic acid into hyocholic acid. This activity is developmentally dependent giving higher expression in the fetal than the adult pig liver. The basic structure of cholic acid is cholesterol, which is quite different from the metabolites of camelina meal. Glucosinolates of camelina are mainly glucocamelina, which is a methyl-sulfinyldecyl isothiocyanates . The transcriptional activation Cyp8b1 is probably the same mechanism as caused by broccoli. Metabolites of broccoli glucosinolate contain sulforaphane which is a methyl-sulfinylbutane isothiocyanate. Sulforaphane activates phase 2 drug enzymes by the nuclear factor, E2 p-45-related factor 2 (Nrf2) transcription factor [32]. The Nrf2 factor binds the antioxidant response element (ARE) to activate transcription of the respective genes [33]. The same Nrf2 transcription factors have been shown to activate the other phase 2 enzymes induced by camelina meal including the Aldh2, TST and GstM1 [34]. Future trials should examine if camelina thiocyanate and its derivatives are actually inducing the Nrf2 transcription factor and the genes which respond to the ARE, such as the phase 1 enzyme Cyp1A1.

## Conclusions

*Camelina sativa* has high erucic acid and high glucosinolate content but it can be grown on marginal land and has good oil yield. The continued industrial use of camelina oil as a biofuel may provide a cheap source of protein as meal for the pig industry but more study into the nutritional aspects is needed. Recent information on the biological action of glucosinolates and erucic acid has suggested that these are not as detrimental as originally thought [4]. Rats are relatively slow at digesting erucic acid and due to its accumulation in the heart , thought to be cardiotoxic [35] but recent studies have shown that the animals will adjust to the feed and increase erucic acid metabolism within their peroxisomes [36]. Glucoinsolates were thought to be only anti-nutritional but now the research evidence has demonstrated that glucosinolates may act as a chemo-protective, anti-cancer agent [12]. This trial on camelina meal feeding in pigs, indicates a potential anti-carcinogen benefit, through the stimulation hepatic expression of phase 1 and 2 xenobiotic detoxifying enzymes.

## Abbreviations

AldH2: Aldehyde dehydrogenase; CYP8b1: Cytochrome P450 8b1; Gstm5: GlutathioneS- transferase mu 5; PCR: Polymerase chain reaction; TST: Thiosulfate transferase.

## Competing interests

The authors declare that they have no competing interests.

## Authors’ contributions

WJM performed the analysis for gene expression and supported the findings with statistical analysis and was the main author of the manuscript. PD carried out the real-time PCR trials. TM was involved in providing the camelina meal. WC performed the animal trials including the design of the diets. All authors read and approved the final manuscript.
